# Severe Hidradenitis Suppurativa and Subsequent Myelodysplastic Syndrome: A Case Report Highlighting Chronic Inflammatory Burden and Carcinogenic Risk

**DOI:** 10.7759/cureus.102845

**Published:** 2026-02-02

**Authors:** Mariela R Rosas García, Guadalupe Maldonado-Colin, Lucia Achell Nava, Bianka D Barajas, Ramiro Alanis

**Affiliations:** 1 Dermatology Department, Centro Médico Nacional 20 de Noviembre, Instituto de Seguridad y Servicios Sociales de los Trabajadores del Estado (ISSSTE), Mexico City, MEX

**Keywords:** biologic treatment, carcinogenic risk, chronic inflammation, follicular occlusion tetrad, hidradenitis suppurativa (hs), il-17 inhibitor therapy, ixekizumab, myelodysplastic syndrome

## Abstract

Hidradenitis suppurativa (HS) is a systemic inflammatory disease associated with an increased risk of malignancies, in which chronic inflammation and immune dysregulation have been proposed as contributing factors. We report the case of a 22-year-old male with severe follicular occlusion tetrad (Hurley III) since pre-puberty (age 11), who developed severe cytopenias at age 16, subsequently diagnosed with myelodysplastic neoplasm. Despite extreme transfusion dependence (>110 cumulative units of red cells and platelets over six years) and persistent bone marrow failure, treatment with Ixekizumab (anti-IL-17A) achieved significant cutaneous improvement, reducing the International Hidradenitis Suppurativa Severity Score (IHS4) from 21 to 11. This case highlights a possible association between long-standing severe HS and myeloid disease, suggesting a biologically plausible link between chronic cutaneous inflammation and myeloid dysfunction. Although late inflammatory control resulted in meaningful dermatologic improvement, it did not reverse established bone marrow failure, emphasizing the importance of early intervention and hematological surveillance in patients with severe HS.

## Introduction

Hidradenitis suppurativa (HS) is a chronic, recurrent, and systemic inflammatory disease affecting approximately 0.4-1% of the global population [1]. It is characterized by painful nodules, abscesses, sinus tracts, and scarring, predominantly in areas rich in apocrine glands [1]. In its most severe forms, including those encompassed within the follicular occlusion syndrome, which comprises HS, acne conglobata, dissecting cellulitis of the scalp, and pilonidal sinus disease, the systemic inflammatory burden is substantial [2].

Recent studies have demonstrated that patients with HS have a significantly increased risk of developing malignant neoplasms compared with the general population [3,4]. Although squamous cell carcinoma is the most frequently associated malignancy, an association with hematologic neoplasms, such as lymphoma and leukemia, has also been reported [5].

Chronic inflammation and immune dysregulation, mediated by pro-inflammatory cytokines, such as TNF-α and IL-17, have been proposed as contributing factors to genomic instability and malignant transformation [1,6]. The coexistence of severe HS and myelodysplastic syndrome (MDS) in young patients is rare and has been reported mainly in isolated cases and small series, supporting a biologically plausible association between sustained systemic inflammation and myeloid carcinogenesis [5].

## Case presentation

**Figure 1:**
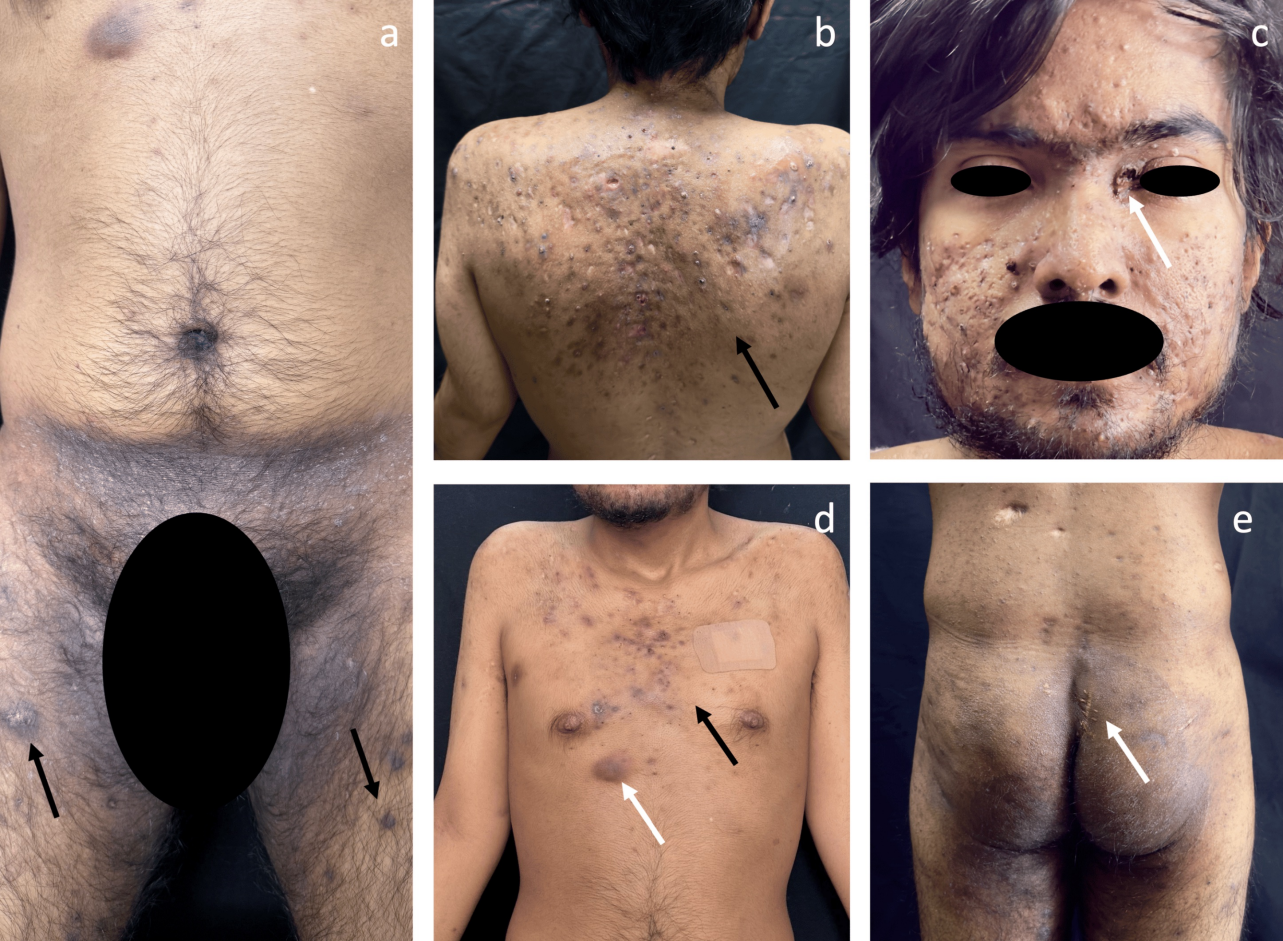
Baseline clinical presentation prior to treatment initiation (a) Inguinal region with active inflammatory nodules (black arrow), erythema, and post-inflammatory hyperpigmentation. (b) Posterior trunk with multiple inflammatory papules and nodules, pustules, and abscesses alternating with ulcers and retractile atrophic scars, consistent with severe disease (Hurley stage III). (c) Facial involvement showing multiple papules, pustules, and inflammatory nodules, an ulcer at the left medial canthus (white arrow), and residual scarring. (d) Anterior trunk demonstrating multiple inflammatory nodules, one active abscess (white arrow), atrophic scars, and post-inflammatory hyperpigmentation. (e) Gluteal region with surgical scar from prior pilonidal cyst excision (white arrow) and areas of post-inflammatory hyperpigmentation.

We present the case of a 22-year-old male patient with a dermatosis characterized by inflammatory follicular lesions involving the trunk and flexural areas (Figure 1), with onset at 11 years of age. Over the following years, the disease progressively evolved into a severe follicular occlusion tetrad (Hurley stage III), comprising HS, acne conglobata, dissecting cellulitis of the scalp (Figure 1c), and pilonidal sinus disease (Figure 1e), refractory to multiple courses of systemic antibiotics. The patient denied tobacco use, alcohol consumption, and illicit drug use.

At the age of 16, the patient developed severe cytopenias and was evaluated by pediatric oncology due to suspicion of juvenile myelomonocytic leukemia. After several years of progressive, severe HS beginning at age 11, hematologic abnormalities first emerged at age 16, preceding the diagnosis of myelodysplastic neoplasm by approximately one year and marking the onset of sustained bone marrow dysfunction. One year later, a bone marrow aspirate confirmed the diagnosis of myelodysplastic neoplasm with dysmegakaryopoiesis and reduced erythroid lineage. Since diagnosis, the patient has remained highly transfusion-dependent, receiving more than 110 units of red blood cells and platelets over a six-year period. Given the overlap between inflammatory bone syndromes and neutrophilic dermatoses, a targeted genetic evaluation was performed, which excluded SAPHO syndrome and did not support an inherited bone marrow failure disorder.

**Figure 2:**
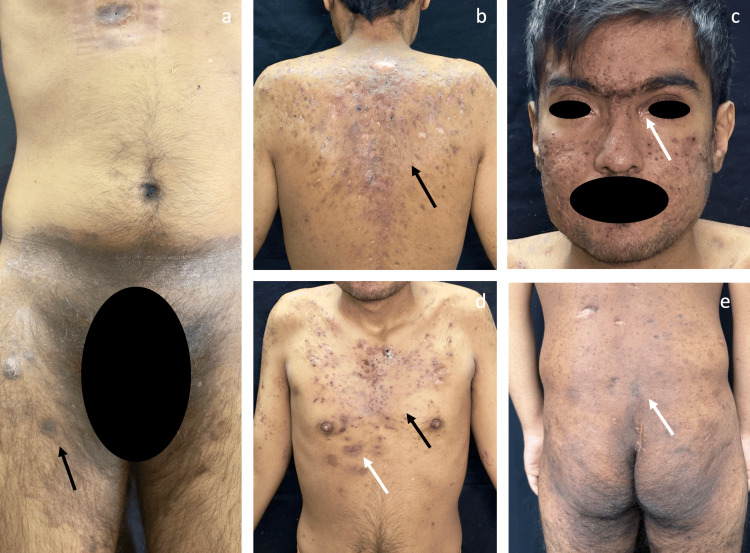
Clinical evolution after completion of the ixekizumab induction phase (a) Inguinal region showing marked reduction of erythema and absence of active inflammatory nodules, with residual post-inflammatory hyperpigmentation. (b) Posterior trunk demonstrating a significant reduction in the number and size of inflammatory lesions, with no active abscesses, and predominance of hyperpigmented macules and residual scarring. (c) Facial involvement with marked decrease in active inflammatory lesions, complete resolution of the ulcer at the left medial canthus (white arrow), persistence of atrophic scars, a few residual papules, and post-inflammatory dyspigmentation. (d) Anterior trunk with clear clinical improvement, absence of active abscesses (white arrow), and decreased inflammatory infiltrate, with persistence of residual scars and macules. (e) Gluteal region without evidence of active inflammation, with residual post-inflammatory hyperpigmentation.

Given the failure of conventional therapies and the severity of HS (baseline IHS4 of 21 and Dermatology Life Quality Index (DLQI) of 18), treatment with ixekizumab (IL-17A inhibitor) was initiated. A favorable cutaneous response was observed at 12 weeks (Figure 2), with improvement in both disease severity and quality of life (IHS4: 11; DLQI: 10). However, persistent bone marrow failure has prompted consideration of adjuvant therapies such as upadacitinib and continuation of the protocol for allogeneic hematopoietic stem cell transplantation.

## Discussion

The relationship between inflammation and cancer is bidirectional and complex. While oncogenic mutations can induce an inflammatory microenvironment (the intrinsic pathway), chronic inflammatory conditions increase the risk of malignancy (the extrinsic pathway) [7]. In HS, immune dysregulation generates a state of sustained systemic inflammation [1], frequently manifesting as part of the follicular occlusion syndrome alongside acne conglobata, dissecting cellulitis, and pilonidal disease [2]. We present a case that illustrates the convergence of these pathways: a young patient with an aggressive myeloid neoplasm and severe cutaneous autoinflammatory disease, supporting a temporal association and a biologically plausible link between long-standing severe cutaneous inflammation and subsequent myeloid neoplasia, highlighting a hypothesis-generating observation.

This case challenges the traditional view of dermatologic comorbidities. We propose that severe HS may represent more than a coincidental condition and could contribute to the biological context in which myeloid disease develops, within a framework described in contemporary oncologic literature.

The extrinsic inflammatory pathway

Mantovani et al. described how chronic inflammation can precede and promote malignant transformation by shaping a pro-tumorigenic microenvironment [7]. In this patient, the sustained release of proinflammatory cytokines from chronically inflamed skin may have contributed to a systemic inflammatory milieu that is biologically compatible with clonal myeloid expansion, rather than indicating a direct causal relationship. In parallel, Greten and Grivennikov have shown that epithelial barrier disruption permits microbial translocation, activating oncogenic signaling pathways in myeloid cells [6]. In HS, follicular rupture and epithelialized tunnels may perpetuate this state of "smouldering inflammation" driven by microbial and immune interactions [1,6].

Genetics and shared molecular pathways

The Notch signaling pathway, whose alterations in the γ-secretase complex are well described in familial HS [8], plays a fundamental tumor suppressor role in epithelial tissues. Although these mutations are less clearly established in sporadic HS, downstream dysregulation of Notch-related signaling may also contribute to carcinogenic susceptibility, as suggested by reports linking HS with squamous cell carcinoma and other malignancies in recent systematic reviews [3,4].

The persistence of HS activity despite neutropenia in this patient is consistent with prior observations in acute myeloid leukemia, suggesting that disease pathogenesis does not rely exclusively on neutrophils but may involve alternative inflammatory and immune pathways [5]. At the molecular level, the IL-23/IL-17 axis represents a central pathway in HS pathophysiology, with IL-17 established as a validated therapeutic target [9]. Emerging data also support the use of biologic agents and targeted therapies to modulate this inflammatory milieu [10]. Together, these observations support a biologically plausible inflammatory link between severe HS and myeloid disease and reinforce the importance of early multidisciplinary evaluation and hematologic surveillance in patients with long-standing, severe HS.

## Conclusions

Severe HS should be increasingly recognized as a systemic inflammatory disorder with potential oncogenic implications. In this case, long-standing severe HS preceded the development of MDS, supporting a temporal association and a biologically plausible link between chronic cutaneous inflammation and premature myeloid oncogenesis. These observations challenge the traditional perception of HS as a localized comorbidity and suggest that, in selected patients, it may function as an active systemic pro-oncogenic condition.

Although late inflammatory control achieved meaningful cutaneous improvement, it failed to reverse established bone marrow damage, underscoring the possible irreversibility of inflammation-driven systemic injury once critical thresholds are exceeded. This case highlights the importance of early risk stratification, close hematologic surveillance, and multidisciplinary management in patients with severe, long-standing HS. Further mechanistic studies are warranted to better define the role of chronic inflammation in carcinogenesis and to guide preventive strategies in high-risk populations.
